# Glycerophospholipid and detoxification pathways associated with small for gestation age pathophysiology: discovery metabolomics analysis in the SCOPE cohort

**DOI:** 10.1007/s11306-020-01740-9

**Published:** 2021-01-05

**Authors:** Aude-Claire Morillon, Debora F. B. Leite, Shirish Yakkundi, Lee A Gethings, Gregoire Thomas, Philip N. Baker, Louise C. Kenny, Jane A. English, Fergus P. McCarthy

**Affiliations:** 1 INFANT Research Centre, Cork University Hospital, Wilton, Cork, Ireland; 2 Department of Obstetrics and Gynecology, University College Cork, Cork, Ireland; 3 Federal University of Pernambuco, Pernambuco, Brazil; 4 Department of Tocogynecology, Campinas’s State University, Sao Paulo, Brazil; 5 Waters Corporation, Wimslow, UK; 6 Manchester Institute of Biotechnology, Division of Infection and Respiratory Medicine, Faculty of Biology, Medicine and Health, University of Manchester, Manchester, UK; 7 SQU4RE, 8800 Roeselare, Belgium; 8 College of Life Sciences, University of Leicester, Leicester, UK; 9 Department of Women’s and Children’s Health, Institute of Translational Medicine, University of Liverpool, Liverpool, UK; 10 Department of Anatomy and Neuroscience, University College Cork, Cork, Ireland

**Keywords:** Small for gestational age infant, Fetal growth restriction, Metabolomics, Lipidomics, Placental insufficiency, Pregnancy complication

## Abstract

**Introduction:**

Small for gestational age (SGA) may be associated with neonatal morbidity and mortality. Our understanding of the molecular pathways implicated is poor.

**Objectives:**

Our aim was to determine the metabolic pathways involved in the pathophysiology of SGA and examine their variation between maternal biofluid samples.

**Methods:**

Plasma (Cork) and urine (Cork, Auckland) samples were collected at 20 weeks’ gestation from nulliparous low-risk pregnant women participating in the SCOPE study. Women who delivered an SGA infant (birthweight < 10th percentile) were matched to controls (uncomplicated pregnancies). Metabolomics (urine) and lipidomics (plasma) analyses were performed using ultra performance liquid chromatography-mass spectrometry. Features were ranked based on FDR adjusted p-values from empirical Bayes analysis, and significant features putatively identified.

**Results:**

Lipidomics plasma analysis revealed that 22 out of the 33 significantly altered lipids annotated were glycerophospholipids; all were detected in higher levels in SGA. Metabolomic analysis identified reduced expression of metabolites associated with detoxification (D-Glucuronic acid, Estriol-16-glucuronide), nutrient absorption and transport (Sulfolithocholic acid) pathways.

**Conclusions:**

This study suggests higher levels of glycerophospholipids, and lower levels of specific urine metabolites are implicated in the pathophysiology of SGA. Further research is needed to confirm these findings in independent samples.

**Electronic supplementary material:**

The online version of this article (10.1007/s11306-020-01740-9) contains supplementary material, which is available to authorized users.

## Introduction

Small for gestational age (SGA) is defined as an infant born with a birthweight less than the 10th percentile when compared to population (weight according to gestational age at birth) or to customised curves (charts individualized on the basis of maternal characteristics) (Sharma et al. 2016b). SGA is associated with placental dysfunction (Dessì et al. 2015) and is a major cause of maternal and neonatal morbidity (Diderholm 2009). Infants born with SGA are at higher risks of perinatal mortality and neonatal complications, such as asphyxia (Rosenberg 2008) and neurological impairment (Grantham-McGregor 1998; Sharma et al. 2016a), as well as longer term morbidity, including increased risk of cardiovascular disorders and type 2 diabetes (Dessì et al. 2012). The lower the birth centile, the higher the risk of short and long term morbidity. SGA can be further classified as moderate (birthweight centile in the 3rd to 10th percentile), and severe (birthweight less than the 3rd percentile) (Lee et al. 2003). A better understanding of the molecular pathways involved in the pathophysiology of SGA could enable better prevention, earlier detection and potential treatment of this pregnancy complication.

Metabolomics is the study of all small weight molecules (50–2000 Da), or metabolites, present in a sample. Metabolic profiling in a clinical research setting has led to the determination of risk factors and pathophysiology of specific diseases (Dunn et al. 2011). Three metabolomics studies using plasma or serum samples showed that the physiopathology of SGA appears to affect lipid and fatty acid metabolism (Horgan et al. 2011; Sulek et al. 2014; Delplancke et al. 2018). In the Greek Rhea cohort study, urine samples taken at 11 weeks of gestation, showed an association between elevated levels of acetate, tyrosine, formate, trimethylamine, lysine and glycoprotein and higher risks of subsequent SGA and preterm birth (Maitre et al. 2014).

To maximise the opportunity to observe metabolic changes that may explain pathophysiological changes seen in SGA, we have analysed maternal plasma and urine. We hypothesised that these changes would precede detection of disease. The aim of our study was to gain further insight into the metabolic and lipidomic pathways involved in the pathophysiology of SGA, using urine and plasma metabolic profiles of women at 20 weeks of gestation, in geographically distinct populations of the SCOPE pregnancy cohort.

## Material and methods

### Participants

**Fig. 1:**
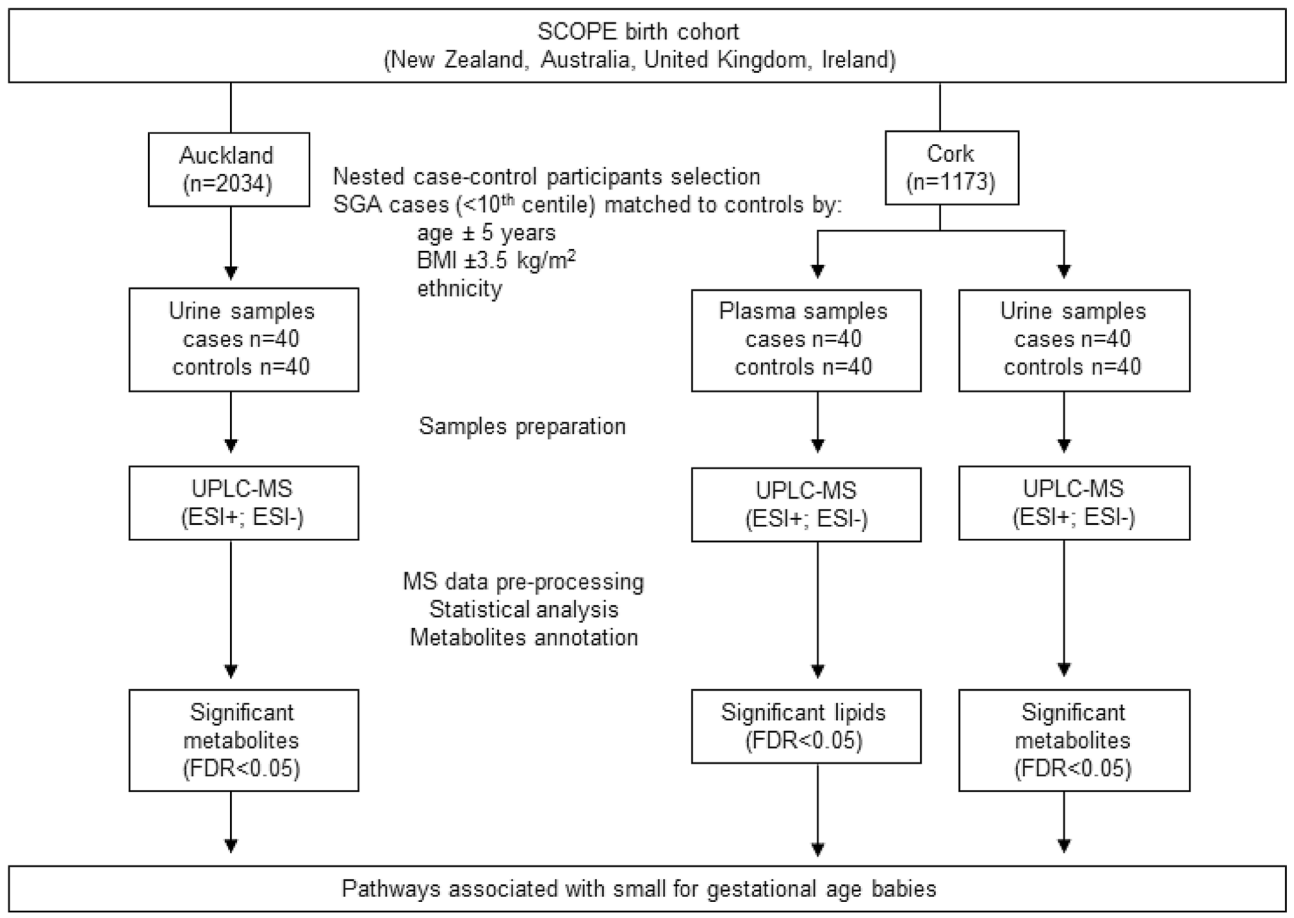
Description of selection of participants, plasma and urine samples preparation and analysis

The present nested case-control study was performed on samples selected from SCreening fOr Pregnancy Endpoints study (SCOPE, www.scopestudy.net) in Cork, Ireland, and Auckland, New Zealand. This study was performed in accordance with the 1964 Helsinki declaration and its later amendments. Informed consent and ethical approval were obtained (Ireland ECM5 (10) 05/02/08, and New Zealand AKX/02/00/364). SCOPE is an international pregnancy cohort that recruited 1773 women in Ireland and 2034 in New Zealand with low-risk and singleton pregnancies (Kenny et al. 2014). Selected participants who delivered small for gestational age babies, with customised birthweights less than the 10th centile (cases), were matched to participants who had healthy and uncomplicated pregnancies (controls). Controls were matched to cases by age (± 5 years), body mass index (BMI ± 3.5 kg/m^2^), and ethnicity (Table 1). For definitions of all clinical endpoints used in Table 1, see Australian New Zealand Clinical Trials Registry (ANZCTR), using study number ACTRN12607000551493. Urine and non-fasted plasma samples were obtained from 40 selected SGA cases in Cork population and matched with 40 controls. In the Auckland population, only urine samples were analysed, with a similar case-control design: 40 women were selected for the SGA group and matched with 40 controls. The nested case-control study designs are summarised in Fig. 1.

**Table 1:** Characteristics of participants and pregnancy outcomes in Auckland and Cork populations

Variables	Auckland			Cork		
	Control (n = 40)	Case (n = 40)	p-value	Control (n = 40)	Case (n = 40)	p-value
Participants characteristics:						
Ethnicity						
Caucasian	35 (87.5%)	35 (87.5%)	1.0	40 (100%)	40 (100%)	NA
Other^a^	5 (12.5%)	5 (12.5%)		0	0	
Socioeconomics index	50 (36.75–50.75)	48 (39.25–59.75)	0.615	43.5 (29.0–50.0)	30 (22.00-45.75)	0.08
Age (years)	31.00 (28.00-32.75)	31.00 (29.00–34.00)	0.448	29 (23.5–31)	28 (24.25-31)	0.412
Primigravida	31 (77.5%)	24 (60%)	0.147^c^	35 (87.5%)	33 (82.5%)	0.755 ^c^
BMI (kg/m^2^) at 15weeks of gestation	23.35 (22.33–26.08)	24.30 (21.68–27.20)	0.969	24.65 (21.58–27.83)	24.85 (23.13–29.38)	0.607
At 20 weeks’ gestation:						
Current smoker	3 (7.5%)	2 (5%)	1.0^c^	4 (10%)	12 (30%)	0.048^c^
Current alcohol consumer	7 (17.5%)	5 (12.5%)	0.755^c^	6 (15%)	6 (15%)	1.0^c^
Current drug user	2 (5%)	1 (2.5%)	1.0 ^c^	0	0	NA
Gestation (weeks)	19.86 (19.18–20.4)	20.14 (19.43–20.43)	0.228	20.86 (20.33–21.14)	20.71 (20.04–21.14)	0.396
Pregnancy outcome:						
GDM						
Yes	0	1 (2.5%)	0.574	0	0	0.31^c^
No	35 (87.5%)	35 (87.5%)		8 (20%)	13 (32.5%)	
Unknown	5 (12.5%)	4 (10%)		32 (80%)	27 (67.5%)	
Diagnosed with PE	0	0	NA	0	0	NA
Diagnosed with GH	0	0	NA	0	1 (2.5%)	1.0^c^
sPTB, < 37w	0	1 (2.5%)	1.0^c^	0	0	NA
Gestational age at delivery (weeks)	40.22 (39.14–41.07)	39.57 (38.9-40.43)	0.214	40.64 (39.9-41.25)	39.86 (38.25-41)	0.014
Customised birthweight centile^b^	54 (30.25–78.75)	3.4 (2–5)	< 0.001	49 (35.45–65.83)	2.35 (0.8–4.48)	< 0.001

Values are shown as mean (SD), median (interquartile range) or n (%)

*NA* Not applicable; *BMI* Body mass index; *GDM* gestational diabetes mellitus; *PE* pre-eclampsia; *GH* gestational hypertension; *sPTB* spontaneous preterm birth; all mothers were nulliparous

^a^Including African, Asian, Maori and Pacific Islander

^b^adjusted for mother’s height, weight at 15 weeks visit, ethnicity, sex and weight of baby and gestation at delivery of baby; ^c^adjusted with Fisher’s Exact Test

Our study included 40 cases and 40 controls with an estimated 95% power to detect small to medium effects. The calculation was performed using MEDCALC® (www.medcalc.com), and showed that assuming a Type I error rate, α, of 5% is sufficient and that will be at least a 50% change in mean (with a similar within sample standard deviation), with the samples size of 40 cases and 40 controls, that the power of the study was 0.9474.

### Reagents and materials

Liquid chromatography (LC) grade methanol, methyl tert-butyl ether (MTBE), acetonitrile (ACN), isopropanol (IPA), formic acid, ammonium formate, and glass tubes were obtained from Fisher Scientific (Loughborough, UK). LC-MS glass vials were purchased from Waters (Waters, Dublin, Ireland).

### Sample preparations

For both Cork and Auckland population, plasma samples were prepared and analysed independently. Cork urine samples were prepared and analysed independently of plasma analysis.

#### Plasma samples

Plasma samples taken at 20 weeks of gestation were prepared in a randomised order. The lipid extraction method used was based on a protocol described by Matyash et al (2008). Plasma samples were taken out of − 80 °C storage and left on ice until thawed; none had been previously thawed. Plasma samples (200 µl) were transferred to glass tubes, pre-chilled methanol (− 20 °C, 600 µl) was added, and vortex mixed for 1 min. MTBE was added (5 ml) to the samples, which were then incubated at room temperature on a shaker for 1 h. Water (1 ml) was added and samples were left on a shaker for 10 min at room temperature; samples were then centrifuged at room temperature for 10 min at 1000 *g*. The organic (top) layer was transferred into glass tubes and left to evaporate overnight, at room temperature. Each sample was reconstituted in 200 µl of 65:30:5, IPA:ACN:Water and vortex mixed for 30 s. Lastly, all samples were transferred to LC glass vials in preparation for LC-MS analysis. Quality control (QC) samples were created by pooling 10 µl from each extracted sample. Multiple aliquots of pooled QC (100 µl per aliquot) were constructed using several LC vials.

#### Urine samples

Urine samples taken at 20 weeks of gestation were prepared in a randomised order, following the Nature Protocol developed by Want et al (2010). Samples stored at − 80 °C, were defrosted on ice, then centrifuged for 10 min at 10,000 *g*. Supernatant (100 µl) was transferred to a new tube and milliQ water with 0.1% of formic acid (FA, 200 µl) was added (ratio 1:2, urine: water 0.1%FA). Samples were then transferred to LC-MS glass vials. Quality control samples (QCs) were prepared by pooling 20 µl from each sample; sample preparation was then the same as for other samples with 100 µl of pooled QC subsequently transferred to several LC vials.

### LC analysis

#### Plasma samples

LC-MS analysis was performed using an ACQUITY ultra performance liquid chromatography (Waters Corp, Milford, MA) coupled with a Synapt G2-S quadrupole time-of-flight (UPLC-Q-TOF) mass spectrometer (Waters Corp, Wilmslow, UK). Samples were analysed on a BEH C_18_ column (1.7 µm, 2.1 × 100 mm) which was maintained at 65 °C, whilst samples were maintained at 7 °C. Analytes were separated over a 23 min gradient using a flow rate of 0.4 mL/min. Mobile phase A was a mix of 10 mM of ammonium formate in acetonitrile:water (ACN:H_2_O, 60:40 (v:v)), and B was a mix of 10 mM of ammonium formate in isopropanol:acetonitrile (IPA:ACN, 90:10 (v:v)). The gradient consisted of initial conditions at 30% of B, before increasing to 99% of B at 18 min and maintaining this for a further 2 min before decreasing to 30% of B over 2 min, returning to initial conditions. Column conditioning consisted of 8 repeat injections of the pooled QC. Samples were analysed as technical triplicates in a randomised order with the pooled QC injected every tenth injection throughout the analysis.

#### Urine samples

LC-MS analysis was performed using an ACQUITY ultra performance liquid chromatography (Waters Corp, Milford, MA) coupled with a Synapt G2-S quadrupole time-of-flight (UPLC-Q-TOF) mass spectrometer (Waters Corp, Wilmslow, UK). Samples were analysed on a BEH C_18_ column (1.7 µm, 2.1 × 100 mm) which was maintained at 40 °C, whilst samples were maintained at 7 °C. Analytes were separated over a 15 min gradient using a flow rate of 0.5 mL/min. Mobile phase A was water and 0.1% formic acid, and B was methanol and 0.1% formic acid. The gradient consisted of initial conditions at 1% of B for 1 min, before increasing to 15% of B over 3 min, increasing to 50% of B over 3 min, and further increasing to 95% of B over 3 min and maintaining this for a further 1 min, before decreasing to 1% of B over 5 min, returning to initial conditions.

Column conditioning consisted of 8 repeat injections of the pooled QC. Samples were analysed as technical triplicates in a randomised order with the pooled QC injected every tenth injection throughout the analysis.

Cork plasma samples were analysed in December 2017 and Cork urine samples in May 2017. Auckland urine samples were analysed in July 2017. All samples were analysed on the same instrument, using a new BEH C_18_ column for each experiment, following the protocols described above.

### MS configuration

For both urine and plasma samples, data were acquired using the data independent acquisition (DIA) mode, MS^E^ (Bateman et al. 2002; Silva et al. 2005) using a Synapt G2-S Q-TOF mass spectrometer (Waters Corp, Wilmslow, UK). Data were acquired in resolution mode, from 50 to 1500 Da, first in positive followed by negative electrospray ionisation modes (ESI+, and ESI−). Both precursor (low energy) and fragment (high energy) ion data were collected during the same acquisition, with 0.1 second scan time for each, and a total cycle time of 0.2 s. A linear collision energy ramp (20–40 eV) was applied for high energy, over the 0.1 second scan. Capillary voltage was set at 3.0 kV, sampling cone at 40 V, extraction cone to 5 V. The source temperature was set at 120 °C, and the desolvation temperature was 650 °C. The desolvation gas flow rate was set at 800 L/h, and cone gas at 50 L/h. Mass calibration was performed using a sodium formate mix (Waters, Wexford, Ireland), recommended by the manufacturer before each batch analysis. Real time lock mass correction was performed using a leucine enkephalin (LeuEnk, 1 ng/µL) mix, injected at 10 µL/min through a lock-spray probe and acquired every 30 s.

### Data processing

Using Progenesis QI version 2.4 (Nonlinear dynamics, Newcastle, UK) was used to align and peak pick the LC-MS data using Progenesis QI automatic processing facility. An appropriate pooled QC was selected by Progenesis QI software, from all the QC samples analysed in the same analysis the automatic algorithm chose the best reference from these runs. This pooled QC was selected as the alignment reference to chromatographically align the data. Data were then peak picked, and considered the adducts corresponding with M + H, M + H–2H_2_O, M + NH_4_, M + Na, M + K, M + 2H, M + 2Na (ESI+), or M–H, M–H_2_O–H, M–Na–2H, M + K–2H, M + Cl, M–2H (ESI−). Downstream statistical analysis was performed using the compound measurements exported from Progenesis QI.

### Statistical analysis

Statistical analysis of the demographics and clinical data was performed using Student T test, Mann-Whitney U test, or Pearson χ^2^ test, with multiple testing corrections as appropriate (IBM SPSS Statistics 24). Results were considered statistically significant at p < 0.05 (Table 1). Before sample preparation and analysis, a block randomisation based on the patients’ BMI and the outcome was performed. No significant dependency between measurement order, the outcome and biometric and clinical information about patients was observed using Mann Whitney U test, Spearman correlation, Chi square test and Kruskal-Wallis test as applicable; Benjamini and Hochberg procedure was applied for multiple testing correction (Benjamini and Hochberg 1995).

Statistical analyses of the UPLC-MS data were performed using the R statistical software(R Core Team 2013) and the Bioconductor package limma (Ritchie et al. 2015), and package ggplot2 (Wickham 2011) was used to create volcano plots. Data obtained from analyses of urine and plasma samples were subjected to the same statistical analyses performed independently. Data were acquired in positive and negative electrospray ionisation modes (ESI+, ESI−), which are known to show significant differences, and were therefore analysed independently. Median normalisation of the raw UPLC-MS data, and application of quality control procedures (Broadhurst et al. 2018) were performed. Measurement precision of each feature was checked by computing coefficient of variation (CV) and the missing rate over the replicate measurements, using pooled QC samples as reference. Features with a missing rate greater or equal to 20% and features with a CV greater or equal to 30% were filtered out. Robust multi-variable methods (Wang et al. 2018; Brereton and Lloyd 2014) were used to rank and select features. Empirical Bayes was used on each feature, and the design was adjusted for replicate measures (Smyth 2004); features analysed were ranked by adjusted p-value. In addition, the Mann Whitney U test was performed on the average measurement per patient with multiple testing correction (Benjamini and Hochberg 1995). Agreement between the two methods (similar trends and results) was shown and confirmed the robustness of the results (Data not shown). The features of interest were tested for correlation with clinical variables, using Wilcoxon-Mann-Whitney Test, Spearman Correlation Test, or Kruskal-Wallis Test as appropriate. Multiple testing correction (Benjamini and Hochberg 1995), with a false discovery rate (FDR) cut-off of 25%, was applied.

### Metabolites putative annotation

In accordance with the MSI reporting standards, we have achieved metabolite identification level 2, or putatively annotated compounds (Sumner et al. 2007). For each dataset, the exact mass of significant features (adjusted p-value < 0.05) were searched against Human metabolome database (HMDB, version 4.0) (Wishart et al. 2018) and Lipidmaps (version of January 2019) (Cotter et al. 2007) using Progenesis QI identification tool. The search was performed using the theorical fragmentation MetaScope mode, which compared our experimental fragments to theorical fragmentation patterns generated by the simulated breaking of bonds in the structures of possible identifications. The search parameters were set for an exact mass tolerance of 5 ppm for the precursor ion, and of 10 ppm for the fragment ion. With UPLC-MS, it is common that metabolites are detected multiple times due to fragmentation, dimerization, chemical adduction, or multiple charging. Putative annotations were reported for unique metabolites, after removing duplicates and metabolites of drugs or metabolites originating from food, and after checking the retention time. For each database identification match, the retention time of the feature was checked to ensure it was in the appropriate time window for the chromatographic separation method used. For instance, if a feature with a retention time of 11 min matched with a Lysophosphatidylcholine (LPC), which are expected to elute early (around 1–2 min) due to their polarity, then it was assumed the feature was not an LPC. Using HMDB, the metabolites reported were grouped into chemical classes.

## Results

### Global lipidomic analysis of plasma samples

Clinical and demographics data from selected Cork SGA cases (customised birthweight < 10th centile, and median 2.35, IQR 0.8–4.48, n = 40) and matched controls are presented in Table 1. Cases were matched to controls by ethnicity, BMI and age and no significant differences were observed in these parameters. Smoking status was significantly different between cases and controls, as was gestational age at delivery (mean 39.19 (SD 2.84) vs. 40.41 (SD 0.94) weeks gestation; p = 0.007).

**Fig. 2:**
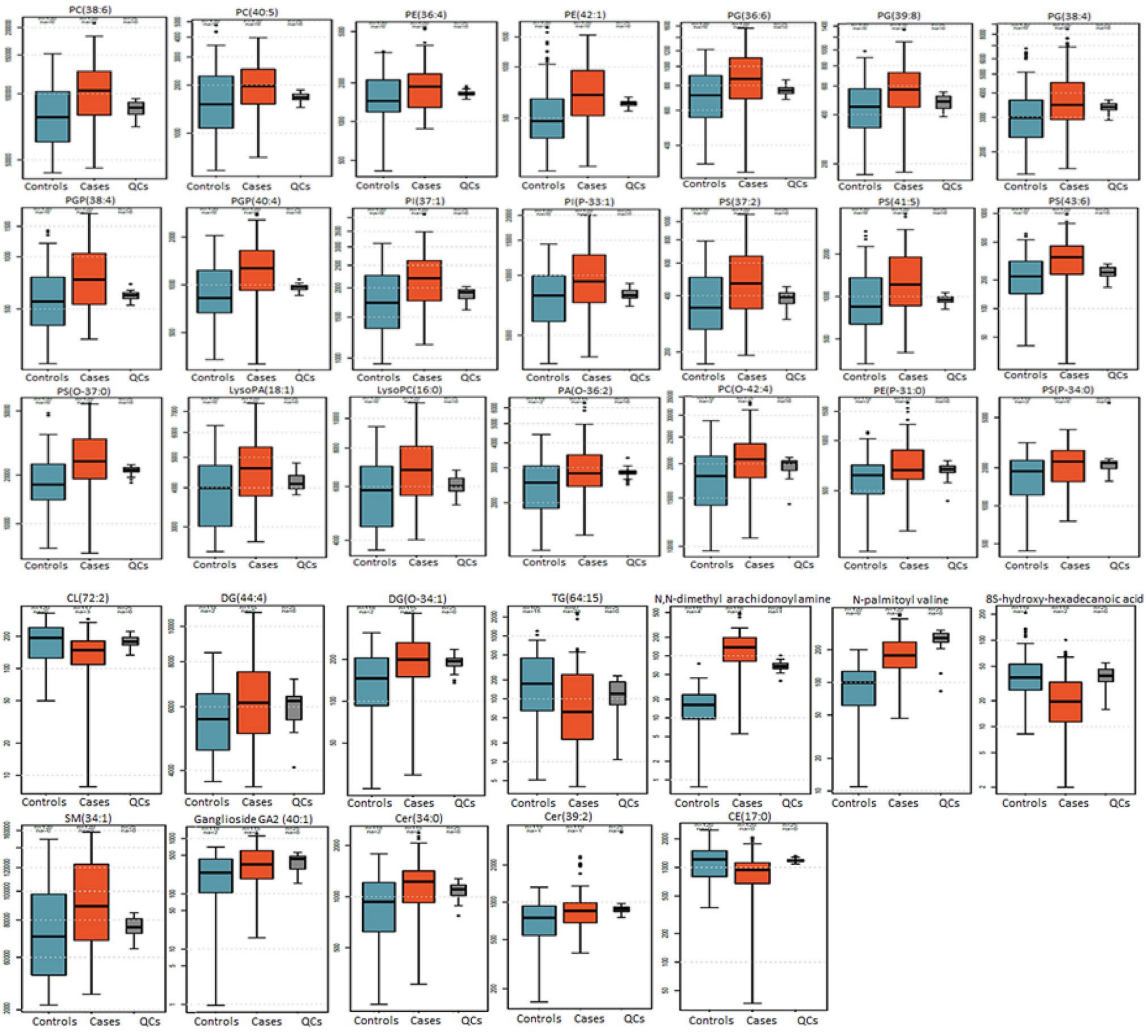
Box plots showing the normalised intensity of features significantly altered (adjusted p-value < 0.05) in small for gestational age group (cases, orange box) compared to control group (blue box), from untargeted UPLC-MS analysis of SCOPE Cork plasma samples (cases n = 40, controls n = 40). Quality control (QCs) group also shown (grey box)

In plasma samples, 8328 (ESI+) and 6,842 (ESI−) features were detected. After normalisation and data filtration, 6179 (ESI+) and 5,048 (ESI−) features were further analysed, and 1167 (ESI+) and 788 (ESI−) features showed adjusted p < 0.05. 33 features (22 in ESI + and 12 in ESI−) were matched to known lipids and are reported (Table 2). Among the 33 lipids putatively annotated, 22 were glycerophospholipids (GPL), including 3 phosphatidylethanolamines (PE), 5 phosphatidylserines (PS), 3 phosphatidylcholines (PC) and 1 lysoPC, 1 phosphatidylglycerophosphate (PA) and 1 lysoPA, 2 phosphatidylinositols (PI), 2 phosphatidylglycerophosphates (PGP), 3 phosphatidylglycerols (PG), and 1 cardiolipin (CL). Except for CL(72:2), all these glycerophospholipids were detected at higher levels in cases when compared to controls. In addition to the glycerophospholipids, 4 sphingolipids were putatively annotated: 2 ceramides (Cer), 1 ganglioside, and 1 sphingomyelin (SM). These 4 sphingolipids were detected in higher levels in cases compared to controls. Finally, 3 fatty acyls, 3 glycerolipids, one organooxygen compound, and one cholesterol fatty ester were identified in Cork plasma samples (Table 1; Fig. 2). Box plots showing the distribution of the lipids of interest in controls (n = 40), cases with a customised birthweight centile below the 3rd (n = 22), and cases with a customised birthweight between the 3rd and 10th centile (n = 18) are presented in Supplementary File 1.

**Table 2:** Lipids that showed significant (adjusted p-value <0.05) differences in SGA group compared to controls in Cork plasma samples

Lipids	Chemical class	Adjusted p-value	Fold change	Direction in SGA group
PE(P-31:0)	Glycerophospholipids	2.89E − 03	1.17	UP
PE(42:1)	Glycerophospholipids	1.23E − 04	1.36	UP
PE(36:4)	Glycerophospholipids	9.68E − 04	1.18	UP
PS(O-37:0)	Glycerophospholipids	2.55E − 06	1.39	UP
PS(41:5)	Glycerophospholipids	1.60E − 04	1.36	UP
PS(37:2)	Glycerophospholipids	1.62E − 04	1.29	UP
PS(43:6)	Glycerophospholipids	4.64E − 04	1.54	UP
PS(P-34:0)	Glycerophospholipids	1.37E − 02	1.22	UP
PC(O-42:4)	Glycerophospholipids	1.24E − 02	1.15	UP
PC(40:5)	Glycerophospholipids	9.68E − 04	1.25	UP
PC(38:6)	Glycerophospholipids	6.68E − 05	1.25	UP
LysoPC(16:0)	Glycerophospholipids	8.57E − 04	1.15	UP
PA(O-36:2)	Glycerophospholipids	3.17E − 02	1.16	UP
LysoPA(18:1)	Glycerophospholipids	7.53E − 04	1.15	UP
PI(37:1)	Glycerophospholipids	1.99E − 04	1.20	UP
PI(P-33:1)	Glycerophospholipids	3.51E − 04	1.23	UP
PGP(38:4)	Glycerophospholipids	1.13E − 05	1.35	UP
PGP(40:4)	Glycerophospholipids	4.87E − 05	1.35	UP
PG(36:6)	Glycerophospholipids	5.94E − 04	1.23	UP
PG(39:8)	Glycerophospholipids	6.25E − 04	1.29	UP
PG(38:4)	Glycerophospholipids	6.43E − 04	1.20	UP
CL(72:2)	Glycerophospholipids	9.75E − 04	0.76	DOWN
DG(44:4)	Glycerolipids	4.25E − 02	1.12	UP
DG(O-34:1)	Glycerolipids	1.73E − 02	1.36	UP
TG(64:15)	Glycerolipids	4.84E − 04	0.47	DOWN
N,N-dimethyl arachidonoyl amine	Fatty acyl	6.69E − 19	7.86	UP
N-palmitoyl valine	Fatty acyl	8.58E − 11	1.99	UP
8S-hydroxy-hexadecanoic acid	Fatty acyl	4.93E − 04	0.53	DOWN
SM(34:1)	Sphingolipids	6.30E − 05	1.25	UP
Ganglioside GA2 (40:1)	Sphingolipids	2.77E − 02	1.56	UP
Cer(34:0)	Sphingolipids	3.30E − 02	1.26	UP
Cer(39:2)	Sphingolipids	3.77E − 02	1.16	UP
CE(17:0)	Steroids and steroid derivatives	3.01E − 04	0.78	DOWN

*CE* cholesterol fatty ester; *Cer* Ceramide; *CL* Cardiolipin; *DG* diglyceride; *LysoPA* Lyso- Phosphatidylglycerophosphate; *LysoPC* Lyso-phosphatidylcholine; *LysoPE* Lyso-phosphatidylethanolamine; *PA* Phosphatidylglycerophosphate; *PC* Phosphatidylcholine; *PE* Phosphatidylethanolamine; *PG* Phosphatidylglycerol; *PGP* Phosphatidylglycerophosphate; *PI* Phosphatidylinositol; *PS* Phosphatidylserine; *SM* Sphingomyeline; *TG* triglyceride

In addition, we determined whether there was a correlation between the lipids of interest and either smoking status, or gestational age at delivery. None of the lipids of interest correlated significantly with gestational age at delivery. However, one lipid was significantly associated with maternal smoking status: CL(72:2) (p-value = 5.82 × 10^−4^ with FDR = 0.06) and was excluded from our results. Box plots showing the levels of this lipid in smokers and non-smokers are shown in Supplementary File 2.

### Global metabolomics analysis of urine samples

Clinical data and demographics of selected women are described in Table 1. Importantly, no significant correlations were found between the metabolites of interest and either smoking status, or gestational age at delivery, in either the Cork or Auckland populations.

In Auckland, urine metabolite profiles detected 11,488 (ESI+) and 10,513 (ESI−) features. After data normalisation and filtration, 11,213 (ESI+) and 10,216 (ESI−) features were further analysed, and 681 (ESI+) and 948 (ESI−) features showed significant differences between cases and controls (adjusted p < 0.05). After a database search, as described in methods section, 3 features (2 in ESI+, 1 in ESI−) matching with known metabolites are reported (Table 3): 1 glucuronic acid (D-glucuronic acid), 1 organooxygen compound (beta-1,4-Mannosyl-N-acetylglucosamine), and 1 steroid derivative (18-Hydroxycortisol). These metabolites were detected in lower levels in cases compared to controls.

**Table 3:** Significant (adjusted p-value <0.05) metabolites of interest detected in urine samples

Metabolite	Chemical class	Biological function or pathway	Adjusted p-value	Fold change	Direction in SGA group	Population
Sulfolithocholic acid	Bile acid	Nutrient absorption and transport Lipid metabolism pathway	7.66E − 03	1.12	Down	Cork
Estriol-16-Glucuronide	Steroid and steroid derivatives	Detoxification Lipid metabolism pathway	3.83E − 04	1.12	Down	Cork
4-Hydroxybenzaldehyde	Hydroxybenzaldehyde		4.79E − 02	1.17	Down	Cork
Neuromedin N (1–4)	Carboxylic acids and derivatives, neuropeptide		2.71E − 02	1.02	Down	Cork
D-Glucuronic acid	Glucuronic acid	Detoxification	4.10E − 02	1.05	Down	Auckland
18-Hydroxycortisol	Steroid and steroid derivatives	Lipid metabolism pathway	4.36E − 02	1.13	Down	Auckland
Beta-1,4-Mannosyl-N-acetylglucosamine	Organooxygen compounds		3.88E − 02	1.05	Down	Auckland

In Cork, urine metabolite profiles detected 16,008 (ESI+) and 8853 (ESI−) features. After data normalisation and filtration by CV and missing rate, 15,468 (ESI+) and 8838 (ESI−) features were further analysed, and 271 (ESI+) and 866 (ESI−) features showed significant differences between cases and controls (adjusted p < 0.05). After a database search, as described in methods section, 4 features (1 in ESI+, 3 in ESI−) matching with known metabolites are reported (Table 3): 1 bile acid (sulfolithocholic acid), 1 steroid derivative (estriol-16-Glucuronide), 1 benzaldehyde (4-Hydroxybenzaldehyde), and 1 neuropeptide (Neuromedin N (1–4)). All four were detected in lower levels in cases compared to controls.

Plasma and urine samples from the same Cork participants were analysed. However, no common metabolite features were identified in both urine and plasma.

## Discussion and conclusions

This study has highlighted the following main findings: (i) altered glycerophospholipids and sphingolipids in plasma samples from women with SGA pregnancies, and (ii) lower levels of metabolites involved in nutrient transport and detoxification pathways in urine samples from women with SGA pregnancies. There were no common SGA-associated metabolite changes identified in plasma and urine.

Our lipidomics study shows evidence of altered glycerophospholipids (GPL) and sphingolipid pathways associated with SGA at 20 weeks’ gestation in Cork plasma samples. Phospholipids are the main lipid class present in biological bilayer, such as cell membranes, and are involved in inflammation, apoptosis, and storage and breakdown of lipids for energy (Baig et al. 2013). Our findings are in agreement with a metabolomics study performed using samples from the SCOPE study taken at 15 weeks of gestation in Australian participants (Horgan et al. 2011). In the Horgan et al. study, metabolic profiles of maternal plasma, venous cord blood, and plasma samples from a rat model of SGA (reduced uterine perfusion pressure, RUPP, model) were obtained on a UPLC system coupled to a hybrid LTQ-Orbitrap mass spectrometer. Horgan et al. identified nineteen metabolites as statistically different in SGA cases as compared to controls; these included sphingolipids and phospholipids, such as several lysophosphatidylcholines and phosphatidylcholines, most of which were detected in higher levels in SGA groups in maternal plasma samples, thereby supporting our findings of increased plasma glycerophospholipids (GPL) and sphingolipids in SGA. In addition, a recent study combining the use of direct infusion MS/MS, LC-MS/MS, proton nuclear magnetic resonance (NMR) and artificial intelligence showed alteration of several pathways in cord blood samples, including phospholipid biosynthesis and fatty acid metabolism, to be associated with SGA (Bahado-Singh et al. 2019). Other pregnancy complications have been associated with altered lipid levels when compared to non-complicated pregnancy, including spontaneous preterm birth (sPTB) (Morillon et al. 2020), recurrent miscarriage and pre-eclampsia (Baig et al. 2013). An animal model of pregnancy loss, where sphingosine kinase, an enzyme part of the sphingolipid pathway, was inactivated showed an increased rate of early pregnancy loss compared to wild type mice (Mizugishi et al. 2007). This further shows the critical role of lipids in the pathophysiology of pregnancy complications.

Two fatty acyls were detected in higher levels in SGA compared to control group, N,N-dimethyl arachidonoyl amine (adjusted p-value = 6.69 × 10^–19^; fold change FC = 7.86), N-palmitoyl valine (adjusted p-value = 8.58 × 10^–11^; FC = 1.99) and on in lower levels, 8S-hydroxy-hexadecanoic acid from Table 2 (adjusted p-value = 4.93 × 10^− 4^; FC = − 1.88). To date, the precise role of these molecules remains unclear, however, they seem to be greatly altered in pregnancy complicated by SGA when compared to uncomplicated pregnancy.

Our metabolomics study of urine samples taken at 20 weeks’ gestation, showed that the metabolites altered in SGA were D-Glucuronic acid in Auckland samples, and Estriol-16-glucuronide in Cork samples both of which are involved in detoxification, and Sulfolithocholic acid is implicated in nutrient absorption and transport. None of the significantly different metabolites of interest were found in both the Cork and Auckland populations, however, several are involved in similar pathways and cellular processes. This may be attributed to the fact that this independent study was run months apart, on a different BEH C_18_ column therefore chromatographic reproducibility was not minimised. Future projects will take a more targeted approach to metabolomics validation given the inherent problems of chromatographic reproducibility.

Overall, the lipidomics and metabolomics analyses performed on samples taken from Cork women suggested placental insufficiency and inadequate transport of lipids to the placenta, resulting in impaired fetal growth (Zhang et al. 2015) detected as early as 20 weeks of gestation. Indeed, the placenta plays a key role in the development of the fetus *in utero*, as it ensures the fetus receives sufficient nutrients—especially oxygen amino acid, glucose and fatty acids (Lager and Powell 2012).

Our study had limitations; one of them was the number of smoking women in Cork population (30% in the SGA group, and 10% in the control group), and another was the significant difference of gestational age at delivery between SGA group and controls in Cork population. This latter difference could be attributed to the fact that the participants selected for the SGA group delivered babies with extremely low customised birthweight centile (median of 2.35, IQR 0.8–4.48) compared to the control group (median 49, IQR 35.45–65.83). Cases and controls were matched for age, ethnicity and BMI when the studies were designed, and exclusion of participants would have reduced the power of this study. In addition, using all known risk factors associated with SGA to adjust the data during statistical analysis would have led to overfitting the data. We did, however, use a stringent false discovery rate cut-off at 5% to select metabolites and lipids of interest, and we further tested the metabolites and lipids of interest to determine if they were significantly correlated with clinical factors (in addition to SGA). No significant correlation was observed with any metabolite of interest and gestational age at delivery, or smoking status, and just one lipid of interest, CL(72:2), was found to be significantly correlated with smoking status. However, CL(72:2)was also significantly correlated with SGA and further analysis to decipher the biological link between CL(72:2) and smoking in preclinical models is warranted to rule out this confounding result. Another limitation of our lipidomics study is that no lipid standards were used to confirm the identities, however library search using accurate mass and fragment ion was performed, thus achieving metabolite identification level 2 according to the MSI reporting standards (Sumner et al. 2007).

In conclusion, this study showed that higher levels of glycerophospholipids and sphingolipids at 20 weeks of gestation, is associated with the onset of SGA in participants of the SCOPE study in Cork. However, whether the correlations represent a cause, or an effect of SGA needs to be further investigated. Further studies are needed to validate these findings in an independent pregnancy cohort and to examine whether there may be the potential to use these lipids to predict pregnancies at risk of SGA.

## Electronic Supplementary Material

Below is the link to the electronic supplementary material.

Supplementary file1 (DOCX 352 kb)

Supplementary file1 (DOCX 23 kb)
