# Supplementary material for: Glycerophospholipid and detoxification pathways associated with small for gestation age pathophysiology: discovery metabolomics analysis in the SCOPE cohort
Source: Metabolomics. 2021 Jan 5;17(1):5. doi: 10.1007/s11306-020-01740-9 (PMC7782411; doi:10.1007/s11306-020-01740-9)
Supplement: Supplementary file 2 — Supplementary file1 (DOCX 23 kb) [file 11306_2020_1740_MOESM2_ESM.docx]

Metabolomics Journal

Glycerophospholipid and Detoxification Pathways Associated with Small for Gestation Age Pathophysiology: Discovery Metabolomics Analysis in the SCOPE Cohort.

Aude-Claire Morillon^1,2^; Debora F. B. Leite^3,4^; Shirish Yakkundi^1,2^; Lee A Gethings^5,6^; Gregoire Thomas^7^; Philip N. Baker^8^; Louise C. Kenny^9^; Jane A. English^1,10,#^; Fergus P. McCarthy^1,2, #^*

^1^ INFANT Research Centre, Cork, Ireland;

^2^ Department of Obstetrics and Gynecology, University College Cork, Cork, Ireland;

^3^ Federal University of Pernambuco, Pernambuco, Brazil;

^4^ Department of Tocogynecology, Campinas’s State University, Sao Paulo, Brazil;

^5^ Waters Corporation, Wimslow, UK;

^6^ Manchester Institute of Biotechnology, Division of Infection and Respiratory Medicine, Faculty of Biology, Medicine and Health, University of Manchester, Manchester, UK;

^7^ SQU4RE, 8800 Roeselare, Belgium;

^8^ College of Life Sciences, University of Leicester, Leicester, UK;

^9^ Department of Women’s and Children’s Health, Institute of Translational Medicine, University of Liverpool, Liverpool, UK

^10^ Department of Anatomy and Neuroscience, University College Cork, Cork, Ireland.

^#^Authors contributed equally.

*Corresponding Author: Fergus P. McCarthy, INFANT Research Centre, Cork University Hospital,

Wilton, Cork, Ireland. Email: Fergus.mccarthy@ucc.ie; Tel: +353 (0)21 4205082


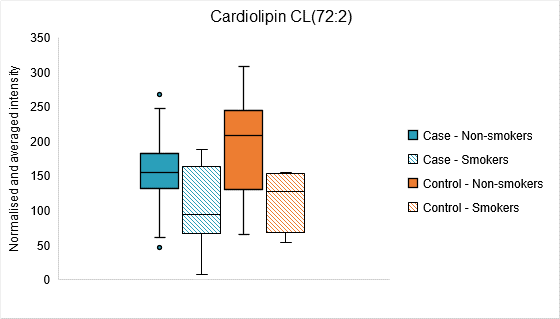


**Online Resource 2** Box plot showing the normalised and averaged intensity of cardiolipin CL(72:2) in four groups from untargeted UPLC-MS analysis of SCOPE Cork plasma samples. Cases are small for gestational age (SGA, customised birthweight ≤10^th^ centile). Blue box: case non- smokers (n=28), Blue pattern box: case smokers (n=12), Orange box: control non-smoker (n=36), and Orange pattern box: control smokers (n=4).
